# QUANTITATIVE RE-IMAGINING OF LTC : RESULTS FROM ROUNDS 1 AND 2 OF A NATIONAL DELPHI STUDY

**DOI:** 10.1093/geroni/igz038.2337

**Published:** 2019-11-08

**Authors:** Ellen McCreedy, Caleb Hoover

**Affiliations:** 1 Brown University, School of Public Health, Providence, Rhode Island, United States; 2 Hennepin County Medical Center, Mineapols, Minnesota, United States

## Abstract

At Round , 110 participants answered an open text question about how they would redesign LTC if starting fresh without regulatory or financial constraints.. They also rated a list of values as to whether they were reflected in the respondents’ suggestions. From analysis of Round 1 open text, principles for LTC were extracted and 20 programmatic building blockscreated. At Round 2, respondents rated the importance of the original value list (after Round 1 results were shared) and rated the principles and building blocks. This paper presents those findings and highlights inconsistencies in results: for example, both a universal LTC and a means-test benefit were endorsed. Participants preferred the term Long-Term Services and Support for the subject matter but no strong term was the favorite for service users. Alternate ways of displaying endorsement of individual items (mean value, different score cutoffs) resulted in the same most popular and least popular items.

